# Efficient Long-term CD20-targeted Therapy for Relapsing Lymphoproliferative Bowel Disease in an AID-deficient Patient

**DOI:** 10.1007/s10875-026-02074-2

**Published:** 2026-11-12

**Authors:** Marcel Horning, AID study group, Marco Fischer, Vasil Toskov, Sarah Salou, Gabriella Cericola, Maria Elena Maccari, Christoph Schell, Christine Forster, Ulrike Teufel-Schäfer, Stephan Ehl, Carsten Speckmann

**Affiliations:** 1 Division of Pediatric Hematology and Oncology, Children’s Hospital, University Medical Center Freiburg, Faculty of Medicine, University of Freiburg, Freiburg, Germany; 2 Institute for Immunodeficiency, Center for Chronic Immunodeficiency (CCI), Medical Center, Faculty of Medicine, University of Freiburg, Freiburg, Germany; 3 Department of General Pediatrics, Adolescent Medicine and Neonatology, Medical Center, Faculty of Medicine, Children’s Hospital, University of Freiburg, Freiburg, Germany; 4 Institute for Surgical Pathology, Medical Center, Faculty of Medicine, University of Freiburg, Freiburg, Germany; 5 Department of Allergology and Clinical Immunology, Children’s Hospital of Eastern Switzerland, St. Gallen, Switzerland; 6 Division of Hematology/Oncology, Boston Children’s Hospital, Boston, MA USA

## To the Editor

Autosomal recessive deficiency of the enzyme activation-induced cytidine deaminase (AID) is an inborn error of immunity (IEI) which leads to a class-switch recombination defect resulting in a marked reduction of serum IgG and normal to elevated levels of IgM, clinically known as Hyper-IgM syndrome type 2 (HIGM2) [1]. HIGM2 patients typically suffer from severe recurrent infections due to hypogammaglobulinemia, but can also present with various symptoms of immune dysregulation, such as arthritis, hepatitis, autoimmune cytopenia and lymphoid hyperplasia, which was found at variable localizations in up to 75% of the patients [2].

Clinical management is mainly based on immunoglobulin replacement therapy (IGRT), which has been reported to also contribute to the control of inflammatory and lymphoproliferative manifestations. Published data shows complete control of lymphoproliferation under IGRT alone in approximately 75% of the patients [3]. Among the others only one patient required additional immunomodulatory treatment due to lymphoproliferation. In the latter, persisting enlarged mesenteric lymph nodes led to intestinal intussusception and required long-term corticosteroid-therapy [3].

We report our treatment experience in two siblings suffering from HIGM2 with a homozygous variant in *AICDA* Exon 2 (c.22_40del19 p.Arg8Asnfs*19) born to consanguineous parents of Turkish inheritance. The family history was unremarkable for IEI or susceptibility to infections but positive for autoimmune events with the mother having diabetes mellitus and a paternal aunt suffering from Crohn’s disease (CD). Functional analysis of the variant has been previously reported to impact the DNA methylation profile of naïve B cells, thus possibly resulting in impaired negative selection of autoreactive B cells in the germinal center [4].

The male index patient presented with recurrent abdominal pain at 4 years of age (supplementary Table 1). Work-up revealed mesenteric lymphadenitis, ileitis terminalis with epitheloid-cellular granulomas and diffuse follicular hyperplasia with expansion of CD20 + B cells in his colon. Stool calprotectin levels reached a maximum of 616 mg/kg [normal levels < 50 mg/kg]. Based on these findings and family history, a Mendelian form of CD was suspected. In addition to these gastrointestinal manifestations, he also presented with a previous history of bronchopulmonary infections, chronic recurrent otitis and mastoiditis, for which he had undergone surgery. This prompted an immunological evaluation, which identified undetectable IgG, but elevated levels for Immunoglobulin M (332 mg/dl [age-specific normal range 40–160 mg/dl]) eventually leading to the diagnosis of AID deficiency. IGRT was initiated, which reduced susceptibility to infections and also led to complete resolution of the patient’s bowel disease over the past ten years. This is in line with experience in previously published AID patients, in whom continuous IGRT led to complete control of both infections and immune dysregulatory symptoms, i.e. bowel disease [3].

**Fig. 1:**
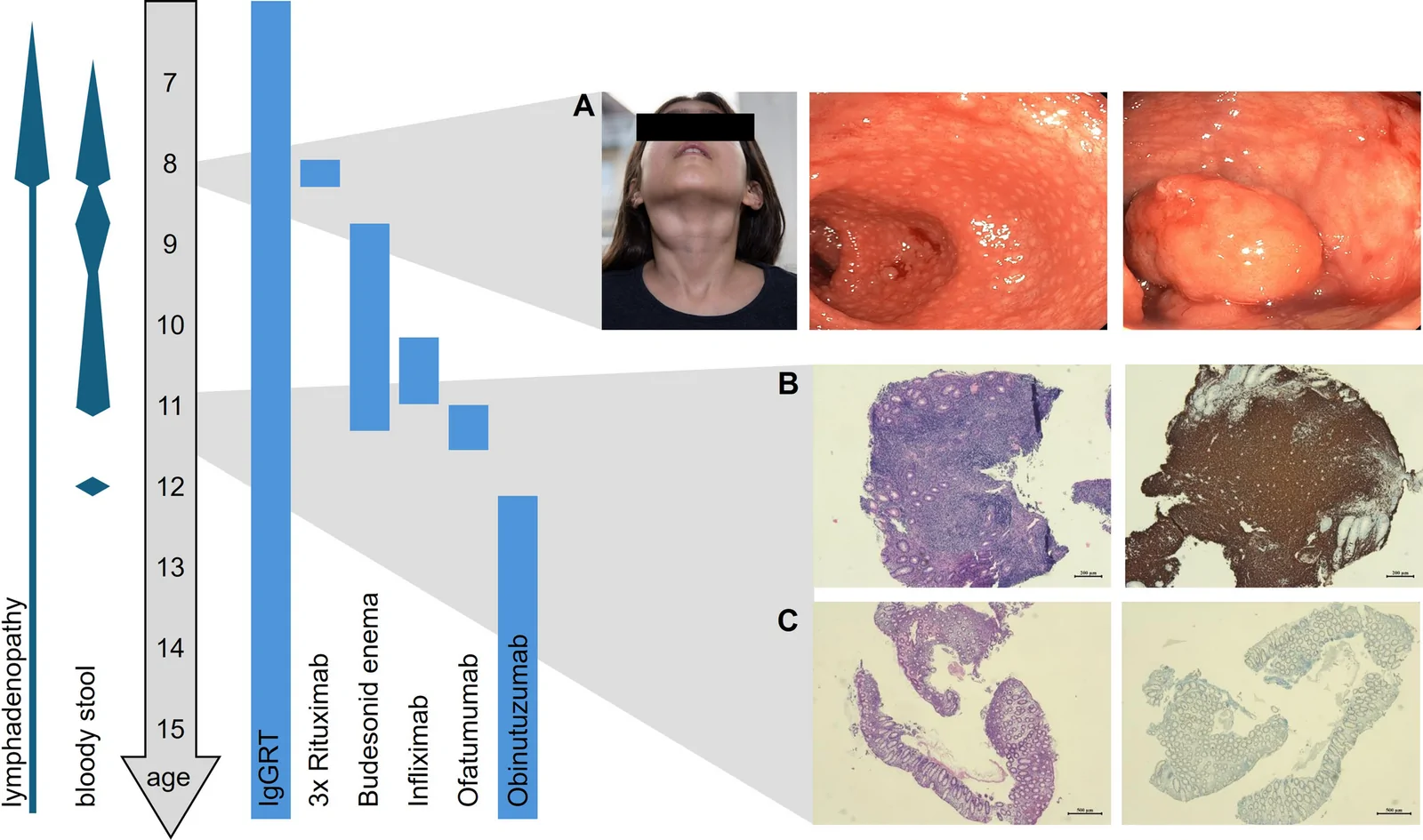
Timeline of clinical symptoms and treatment course since the onset of symptoms at the age of seven. Dark blue markers illustrate the clinical course of the symptoms with the thickness correlating with severity of the symptom at given age. Light blue markers represent the different treatment blocks over time. (**A**) Clinical symptoms (Cervical lymphadenopathy, colonoscopy with prominent lymphofollicular hyperplasia, broad-based rectal polyp) under IGRT and before any CD20 directed treatment. The microscopy slides are rectal biopsies with staining for HE (left) and CD20 (right). They show the recurrence of CD20 predominated polyclonal infiltrates before restarting CD20 directed therapy (**B**), with good response and now absent CD20 positive B cells after 5 doses of ofatumumab (**C**)

Symptom onset in the index patient’s sister was more insidious with recurrent otitis during preschool and moderate failure to thrive (Fig. 1 and Supplementary Table 1). At 7 years of age, she developed chronic productive cough and presented with a first pneumonia prompting immunological evaluation. She had profound hypogammaglobulinemia and was diagnosed with the same homozygous AID variant. Initially, levels for IgA, IgG and IgE were not detectable, while IgM was significantly elevated (> 1000 mg/dl [age specific normal range 40–160 mg/dl]). IGRT was promptly initiated and resulted in good control of her previous infection susceptibility. Yet, six months into IGRT and despite maintenance of high IgG trough levels (IgG > 500 mg/d [age specific normal range 600–1300 mg/dl]), she developed a progressive benign cervical lymphadenopathy and reported intermittent bloody stool. Calprotectin levels in stool exceeded 2000 mg/kg [normal range < 50 mg/kg] and ileocolonoscopy revealed severe benign lymphofollicular hyperplasia in the terminal ileum, distal colon and rectum with CD20 predominant infiltrates. A broad microbiology screening excluded an infectious trigger of her bowel inflammation. At the same time, routine laboratory tests showed a latent autoimmune-thyroiditis with detection of anti-thyroid peroxidase- and anti-thyroglobulin-antibodies.

Treatment with rituximab (375 mg/m² in weekly intervals) was initiated and markedly reduced the lymphoproliferative manifestations and normalization of previously elevated calprotectin levels, but had to be discontinued due to severe anaphylactic reaction, which occurred during the third administration.

Alternative treatment approaches were discussed. Topical budesonide was able to improve, but not completely control gastrointestinal symptoms. Moreover, peripheral lymphadenopathy persisted and any delay in budesonide application led to rapid recurrence of the symptoms. Eventually the patient developed a broad-based polypoid rectal obstruction. A course of anti-TNF-alpha treatment with Infliximab was initiated but had no effect.

Lymphatic hyperplasia reoccurred nine months after the third dose of rituximab. The histology resembled results of the initial biopsies, with expansion of mostly CD20 positive B cells (Fig. 1).

The recurrence of CD20 positive cells together with clinical progression of the gastrointestinal symptoms led to the discussion of other CD20-directed therapeutic options. However, at the time, rituximab was the only CD20 directed antibody licensed for children. Nevertheless, given the good response to rituximab in the past and evidence of a target for a directed therapy, we decided to switch to an alternative off-label anti-CD20 treatment with ofatumumab and later, after its discontinuation on the European market, with obinutuzumab. The administration of obinutuzumab followed an individualized (clinical symptom based) maintenance schedule (1000 mg once a month). This off-label treatment resulted in full clinical and immunohistochemical remission. Obinutuzumab treatment was tolerated well without adverse events. Attempts to withdraw obinutuzumab or to increase dosing intervals resulted in relapse of bowel disease, even though flow cytometry showed a persistent B cell depletion. Consequently, sustained obinutuzumab therapy in monthly intervals, remained indispensable for symptom control in our patient for the last six years. As rituximab was discontinued because of anaphylaxis rather than lack of efficacy, our case primarily demonstrates that sequential anti-CD20 therapy is feasible when rituximab cannot be continued. Although ofatumumab and obinutuzumab differ from rituximab in CD20 epitope recognition and mechanisms of B-cell depletion, whether these differences contributed to the favorable long-term outcome in our patient remains speculative. At the last follow-up at 18 years of age the patient showed excellent symptom control without any signs for malignancy under continuous IGRT and monthly obinutuzumab treatment.

Throughout the years, regular endoscopies with intestinal biopsies were performed to monitor the treatment response and to detect any possible malignant transformation in early stages. Clonality analysis of the IgH heavy chain remained unremarkable over the years.

Hematopoietic stem cell transplantation (HSCT) has been discussed with the family as a potential alternative and curative treatment option but given the potential risk and stable clinical course under anti-CD20 targeted treatment, the family has been hesitant to consider other treatment alternatives.

Overall, our clinical observations demonstrate that immune dysregulation may be the first presenting manifestation in HIGM2 and that different treatment responses may occur in individuals with the same underlying variant. They also show that benign but critical lymphoproliferative manifestation may occur in HIGM2 despite adequate IGRT and that additional treatment may therefore be required. In our patient, B cell-depleting therapy proved highly effective, with sustained disease control achieved through the off-label use of ofatumumab and subsequently obinutuzumab. This case further illustrates that, in selected patients, prolonged and consistent B cell depletion may be required to achieve durable control of severe immune dysregulation. At the same time, it highlights the limitations of conventional anti-CD20 antibody therapy, including the need for repeated administration and potential treatment-limiting hypersensitivity reactions. As novel B cell-targeted approaches such as Bruton tyrosine kinase (BTK) inhibitors and B cell-directed chimeric antigen receptor (CAR) T-cell therapies continue to emerge, they may represent promising future treatment options for carefully selected patients with refractory immune dysregulation in IEIs, extending their application beyond malignant diseases [5].

## Supplementary Information

Below is the link to the electronic supplementary material.

Supplementary Table 1: Clinical presentation and treatment response to IGRT in the two siblings.

## Data Availability

No datasets were generated or analysed during the current study.

## Declarations

### Compliance

The authors affirm that the patient provided informed consent for publication of the images.
