# Supplementary material for: Efficient Long-term CD20-targeted Therapy for Relapsing Lymphoproliferative Bowel Disease in an AID-deficient Patient
Source: J Clin Immunol. 2026 Nov 12;46(1):105. doi: 10.1007/s10875-026-02074-2 (PMC13768239; doi:10.1007/s10875-026-02074-2)
Supplement: Supplementary file 1 [file 10875_2026_2074_MOESM1_ESM.docx]

|  | **Index patient (male)** | **Sister** |
| --- | --- | --- |
| **Age at symptom onset** | 4 years of age | 7 years of age |
| **Initial presentation** | •       Bloody stool | •       Recurrent otitis |
|  | •       Recurrent bronchopulmonary infections | •       Moderate failure to thrive |
|  | •       chronic recurrent otitis and mastoiditis | •       Pneumonia |
|  |  | •       lymphadenopathy |
| **Lymphadenopathy** | Mesenteric lymphadenitis | Cervical lymphadenopathy |
| **Gastrointestinal** | •       ileitis terminalis with diffuse follicular hyperplasia | •       Severe benign lymphofollicular hyperplasia in the terminal ileum, distal colon and rectum |
|  | •       CD20 predominant | •       CD20 predominant infiltrates |
| **Response to IGRT** | +    reduced susceptibility to infections | +    reduced susceptibility to infections |
|  | +    complete resolution of bowel disease | −    progressive lymphoproliferation |
